# The nuclear factor of activated T cells 5 (NFAT5) contributes to the renal corticomedullary differences in gene expression

**DOI:** 10.1038/s41598-022-24237-y

**Published:** 2022-11-24

**Authors:** Dmitry Chernyakov, Annika Fischer, Max Brandau, Federica Petrillo, Robert A. Fenton, Bayram Edemir

**Affiliations:** 1 Department of Medicine, Hematology and Oncology, Martin Luther University Halle-Wittenberg, Ernst-Grube-Str. 40, 06120 Halle (Saale), Germany; 2 Department of Biomedicine, Aarhus University, Aarhus, Denmark; 3 Institute for Physiology, Pathophysiology and Toxicology, Zentrum für Biomedizinische Ausbildung und Forschung (ZBAF), Witten/Herdecke University, Witten, Germany

**Keywords:** Nephrology, Physiology

## Abstract

The corticomedullary osmotic gradient between renal cortex and medulla induces a specific spatial gene expression pattern. The factors that controls these differences are not fully addressed. Adaptation to hypertonic environment is mediated by the actions of the nuclear factor of activated T-cells 5 (NFAT5). NFAT5 induces the expression of genes that lead to intracellular accumulation of organic osmolytes. However, a systematical analysis of the NFAT5-dependent gene expression in the kidneys was missing. We used primary cultivated inner medullary collecting duct (IMCD) cells from control and NFAT5 deficient mice as well as renal cortex and inner medulla from principal cell specific NFAT5 deficient mice for gene expression profiling. In primary NFAT5 deficient IMCD cells, hyperosmolality induced changes in gene expression were abolished. The majority of the hyperosmolality induced transcripts in primary IMCD culture were determined to have the greatest expression in the inner medulla. Loss of NFAT5 altered the expression of more than 3000 genes in the renal cortex and more than 5000 genes in the inner medulla. Gene enrichment analysis indicated that loss of NFAT5 is associated with renal inflammation and increased expression of kidney injury marker genes, like lipocalin-2 or kidney injury molecule-1. In conclusion we show that NFAT5 is a master regulator of gene expression in the kidney collecting duct and in vivo loss of NFAT function induces a kidney injury like phenotype.

## Introduction

Urine-concentration by the mammalian kidney requires the generation of an interstitial osmotic gradient to provide the driving force for water absorption from the renal collecting ducts (CD)^1^. This osmotic gradient is generated by active transepithelial reabsorption of NaCl^1–4^. Osmotic equilibration of the tubule fluid via aquaporin-mediated water transport^5^ into the interstitium, coupled with urea transport mechanisms in the inner medulla (IM)^6^, enables urine osmolality to reach up to 1200 mosmol/kg in human and around 4000 mosmol/kg in mice^7^; reflecting the osmolality of the IM interstitium^8^. Consequently, cells in the IM are faced with a hypertonic environment due to the high luminal NaCl and urea concentration, but also a hypertonic interstitium. On the other hand the unique hypertonic environment controls the expression level of cell and segment specific genes. In primary cultivated inner medullary collecting duct (IMCD) cells, the hypertonicty of the cell culture medium induced the expression of genes like aquaporin-2 (*Aqp2*), the ran binding protein 3 like *(Ranbp3l)* and many others^9^. Gene expression analysis using different nephron segments showed that genes that are up regulated by hypertonicity in IMCD cells showed the highest expression in those segments that are physiologically faced with the hypertonic interstitium^10,11^. Analysis of single cell gene expression (snSeq) from mice kidneys showed that the expression level for example *Aqp2* or *Ranbp3l* is highest in cells that are localized in the inner medullary segments^12^. Another study using snSeq identified a gene expression pattern that is associated with the corticomedullary osmotic gradient^13^. These studies show that the hypertonic environment is not only the driving force to generate a concentrated urine but also controls the segment specific gene expression. However, hypertonic stress for the majority of cells can cause DNA damage and induce cell death^14^ but the cells of the renal medulla have developed mechanisms to adapt and maintain their function. For example, cells accumulate compatible organic osmolytes like taurine, myo-inositol, betaine or sorbitol. This accumulation is mediated by the actions of the myo-inositol transporter (SMIT or *SLC5A3*)^15^, the sodium coupled betaine transporter (BGT1 or *SLC6A12*)^16,17^, the sodium coupled taurine transporter (TauT or *SLC6A12*)^17^ or via enzymes such as aldose reductase (AR)^18^. Hypertonicity also induces expression of heat shock protein 70 (*HSP70*), which in combination with other heat shock family members, protects cells from undergoing apoptosis^19^.

The central hub for the majority of these processes is proposed to be the nuclear factor of activated T cells 5 (NFAT5, also known as tonicity-responsive enhancer-binding factor TonEBP), a transcription factor that is activated under hypertonic conditions, translocates into the nucleus and induces the expression of genes that are involved in the accumulation of organic osmolytes, the expression of *HSP70*^14,20,21^, different urea transporters and the water channel aquaporin-2 (*AQP2*)^22–24^.

Although NFAT5 actions are known to be induced by hypertonicity in the kidney, whether it plays a role independently of hypertonic challenge is unclear and a systematic analysis of NFAT5 mediated gene expression in the kidney or renal cell lines is missing. One possible explanation for this is that global NFAT5 deficient mice show high mortality due to impaired kidney and heart development^25,26^.

In this study, we used RNA-seq to assess gene expression profiles in primary cultivated inner medullary collecting duct (IMCD) cells with in vitro genetic deletion of NFAT5 and in primary IMCD cells isolated from novel collecting duct principal cell (PC) specific NFAT5 deficient mice cultured under control and hypertonic conditions. By correlating the hyperosmotic condition with kidney segmentation, we also analyzed the gene expression in the renal cortex and inner medulla in PC deficient NFAT5 mice. This study represents the first analysis of NFAT5 mediated gene expression in the kidney and underlines the importance of NFAT5 in renal function.

## Results

### Effect of in vitro deletion of NFAT5 in primary cultivated IMCD cells

**Figure 1:**
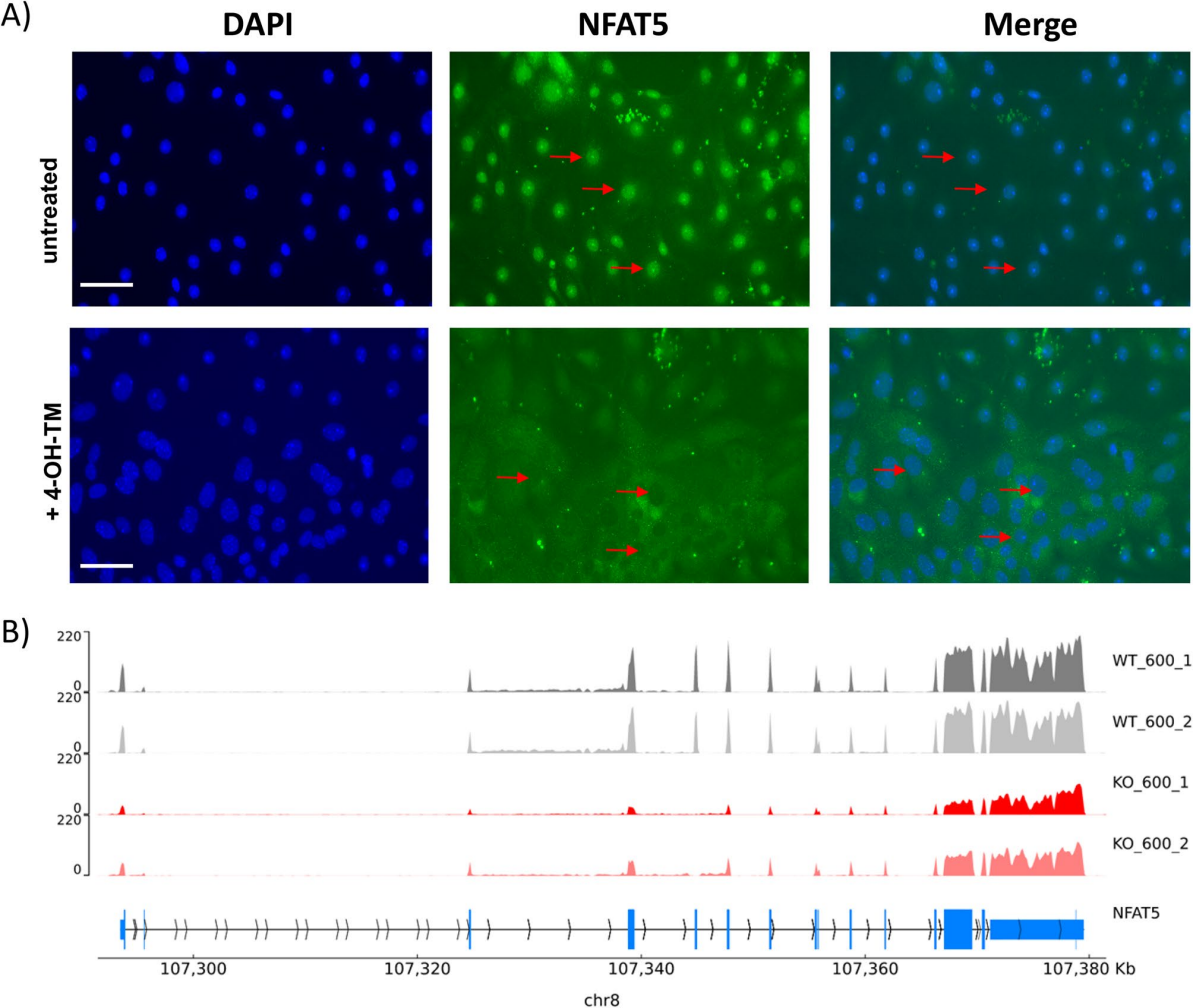
4-Hydroxytamoxifen induces in vitro deletion of NFAT5 expression in primary cultivated IMCD cells. (**A**) IMCD cells were prepared from NFAT5^fl/fl^-Ubc-CreERT^+/−^ mice and cultivated at 600 mosmol/kg. The cells were either left untreated (upper panel) or treated for 24 h with 4-hydroxytamoxifen. After 3 days of additional cultivation, the expression of NFAT5 was analyzed by immunofluorescence using an NFAT5 specific antibody and an Alexa-488 labeled secondary antibody. DAPI was used to stain the nucleus. In untreated cells NFAT5 can be clearly observed in cell nuclei, whereas nuclear labeling is absent in cells treated with 4-Hydroxytamoxifen. Red arrows mark positive and negative NFAT5 nuclei. (**B**) Mapping of the reads from the RNA-seq to the *Nfat5* gene locus using the IGV shows effective deletion of exon 4.

**Figure 2:**
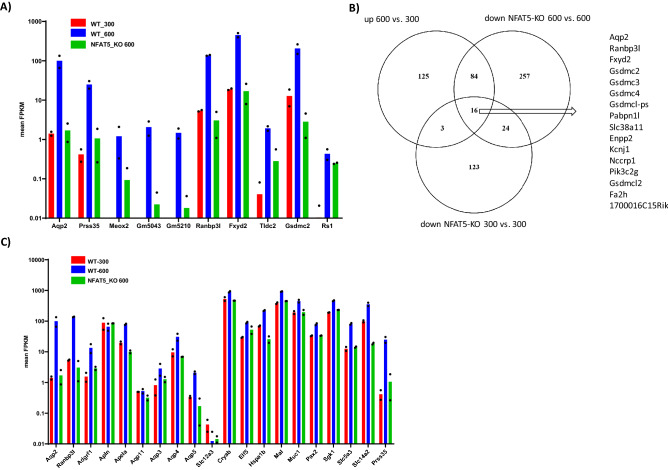
Effect of NFAT5 deletion on top ten hypertonicity induced transcripts. (**A**) Mean FPKM levels of the top ten transcripts that showed the greatest increase under hypertonic (600 mosmol/kg) cell culture compared to control (300 mosmol/kg) conditions in WT IMCD cells. The green bars are showing the mean FPKM level in NFAT5-KO cells cultivated under hypertonic conditions. (**B**) Venn diagram shows common genes up regulated in control cells under hypertonic (600 vs. 300) and down regulated in NFAT5-KO cells under hypertonic (NFAT5-KO 600 vs. 600) and isotonic (NFAT5-KO 300 vs. 300) cell culture conditions. Transcripts with a log2 fold change of at least − 2/2 were included for analysis. The 16 common transcripts are listed on the right side. (**C**) Mean FPKM levels of a set of common hypertonicity induced transcripts and as control of *Apln, Aqp11* and *Slc12a3* in WT control cells cultivated either at 300 (WT_300) or at 600 (WT_600) mosmol/kg. The green bars show the mean FPKM level in NFAT5-KO cells cultivated under hypertonic cell culture conditions (NFAT5_KO-600). For all experiments n = 2.

Primary IMCD cells were isolated^9,27^ from an inducible NFAT5-KO mouse model (Nfat5^fl/fl^-Ubc-CreERT^+/−^) where NFAT5 deletion is mediated by tamoxifen treatment^28^. The successful deletion of NFAT5 was validated by immunofluorescence (Fig. 1A). Total RNA was isolated and processed for RNA-seq to identify hypertonicity-induced changes in gene expression. The mapping of the reads using the integrated genome browser (IGV) again showed that deletion of exon 4 was successful (Fig. 1B). Under hypertonic cell culture conditions, 228 transcripts were increased in expression (log2 fold change > 2) and 318 transcripts reduced (log2 fold change < -2). Figure 2 shows the top 10 upregulated transcripts between control cells cultivated at 300 vs cells cultivated at 600 mosmol/kg based on mean expression levels (complete list including up and down regulated genes are provided as Supplemental Excel file 1). *Aqp2* showed the highest level of induction by hypertonicity, underlining the impact of hypertonicity on *Aqp2* transcriptional regulation. Other induced transcripts include the ran binding protein 3 like (*Ranbp3l*), prolin rich serine protease 35 (*Prss35*) and the FXYD domain containing ion transport regulator 2 (*Fxyd2*); all previously observed as hypertonicity-induced transcripts in rat primary cultivated rat IMCD cells^9^. Next, we compared the changes in expression level of these genes in NFAT5-KO-IMCD cells. Loss of NFAT5 function led to reduced expression of 167 and increased expression of 93 (with a log2 fold change of > − 2/2) transcripts in cells cultivated under isotonic cell culture conditions. Under hypertonic cell culture conditions, loss of NFAT5 reduced the expression of 381 genes, whereas 189 genes were increased relative to control cells (Supplemental Excel file 1). The expression levels of each of the top ten hypertonicity induced transcripts were reduced in NFAT5-KO cells, indicating that NFAT5 directly contributes to their hypertonicity induced expression (Fig. 2A). To identify additional genes that might be regulated by NFAT5 we compared the list of transcripts increased under hypertonic cell culture conditions with those downregulated in NFAT5 deficient cells, both under isotonic and hypertonic cell culture conditions. The Venn diagram in Fig. 2B shows the number of common and unique transcripts. The expression of 100 transcripts are lower in NFAT5 deficient cells under hypertonic cell culture conditions, 16 of which are already reduced under isotonic cell culture conditions. These include transcripts like *Aqp2, Ranbp3l, Fxyd2* and *Gsdmc2-4. Gsdmc* are coding for members of the gasdermin protein family^29^. Interestingly *Gsdmc2, Gsdmc3* and *Gsdmc4* have the highest expression in the PCs of renal IM (Supplemental Fig. 1). The complete list showing common NFAT5 regulated transcripts is provided as supplemental data (Supplemental Excel file 2).

The spatial expression pattern of genes known to be regulated by hypertonicity^9,13^ such as *Aqp2*, *Aqp3, Aqp4, Slc14a2, Apela, Agfrf1* and genes in the proximal tubule e.g. *Aqp11* and *Apln* or the distal tubule e.g. *Slc12a3* were compared in control and NFAT5-KO cells (Supplemental Fig. 2). Small changes in expression were observed for *Apln, Aqp11* and *Slc12a3.* The expression of several common hypertonicity regulated transcripts were induced in primary IMCD cells under hypertonic cell culture conditions and reduced in NFAT5 deficient IMCD cells (Fig. 2C).

### Effect of hypertonicity in primary cultivated IMCD cells with in vivo deletion of NFAT5

The same analysis as described above was performed using a recently generated principal cell (PC) specific NFAT5 deficient mouse by breeding Nfat5^fl/fl^ mice with Aqp2-Cre mice^30,31^. In control cells, hypertonicity induced the expression of *Aqp2, Prss35* and *Ranbp3l* (Supplemental Fig. 3A). Similar to the results obtained by in vitro deletion of NFAT5, IMCD cells prepared from mice with PC specific loss of NFAT5 expression already had an altered gene expression profile when cultivated under isotonic cell culture conditions (Supplemental Excel file 1). Under hypertonic cell culture conditions the expression of hypertonicity induced transcripts were dramatically lower in PC specific NFAT5-KO cells compared to control cells, with *Aqp2* having the highest difference in expression level (Supplemental Fig. 3A). However, the reduction of *Ranbp3l* was not that prominent and *Prss35* and *Fxyd2* expression was even higher. Comparison of the lists of genes (as described above) identified 10 transcripts that are common (increased by hypertonicity in control and reduced in NFAT5 deficient PC cells). These included *Aqp2, Gsdmc2-4* and *Slc38a11,* similar to that observed after in vitro deletion of NFAT5. Novel transcripts included *Adgfr1, Cadps* and *Celf3* (Supplemental Fig. 3B), which also had the highest expression level in the PC of the renal IM (Supplemental Fig. 3C). Together these results highlight the enormous changes in gene expression that occur in primary IMCD cells under hypertonic cell culture conditions. Furthermore, the majority of these changes under in vitro conditions are mediated by the actions of NFAT5, suggesting that NFAT5 is the major driver of hypertonicity-induced gene expression.

### Effect on gene expression in vivo

**Figure 3:**
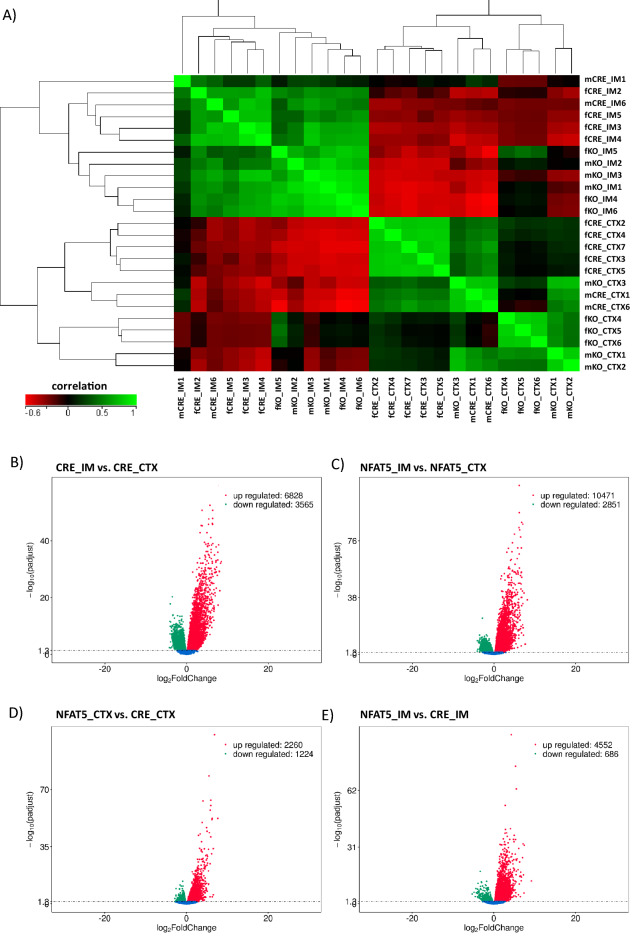
Hierarchical clustering and correlation analysis of transcripts in the renal cortex and inner medulla of control and NFAT5-KO kidneys. (**A**) The global gene expression list from renal cortex (CTX) and inner medulla (IM) of male and female control (CRE) and NFAT5-KO (KO) mice was used for correlation analysis and hierarchical clustering. The color bar indicates the correlation efficiency. The Volcano plots show the number of differentially expressed genes in renal IM (**B**) compared to renal CTX in Aqp2-Cre control mice, (**C**) in IM vs. CTX in PC specific NFAT5-KO mice, (**D**) in CTX of NFAT5-KO mice vs. CTX of control mice and (**E**) in IM of NFAT5-KO mice vs. IM of control mice.

The primary cultured IMCD cells serve as a good model to study physiological signaling pathways in the CD since they endogenously express (hypertonicity induced) key markers of the CD like *Aqp2-4, Slc14a2* and other factors. However, to study the impact of NFAT5 deletion in vivo, we analyzed differences in gene expression in recently developed PC specific NFAT5-KO mice (described above) and Aqp2-Cre^+/−^ mice as controls. The renal phenotype of this novel mouse line includes greatly reduced *Aqp2* expression and nephrogenic diabetes insipidus^31^. To further examine potential sex-differences in NFAT5 function, RNA-seq was performed on RNA isolated from the renal cortex (CTX) or renal inner medulla (including papilla, IM) of male or female mice. Figure 3A shows hierarchical clustering and correlation analysis of the samples. Samples from the same segment clustered together indicating similar expression patterns. Within these segments, male and female samples from control and NFAT5-KO kidneys clustered together, indicating greater sex specific differences relative to the effect of NFAT5 deletion. Other studies describing sex specific differences in gene expression in the kidneys suggest that many of these changes occur in the proximal tubule^12^. Our results support this results, with differences in expression between male and female kidneys being more prominent in the CTX (~ 3900 differentially expressed transcripts) than the IM (only 85 transcripts). The number of sex-dependent differentially expressed genes were also similar in NFAT5 deficient mice (Supplemental Fig. 4).

For the next analysis, we combined male and female samples and analyzed expression differences in the renal CTX vs. renal IM (Fig. 3B). In the renal IM, more than 10,000 transcripts are differentially expressed compared to CTX, with 6828 transcripts of higher expression and 3565 transcripts of lower expression. When we compared the differences in expression in IM vs CTX in NFAT5 deficient mice, more than 13,000 transcripts were differentially expressed (10,471 increased and 2851 decreased, Fig. 3C). Similar to the studies with the primary cultivated IMCD cells, we analyzed the contribution of NFAT5 on gene expression in the renal CTX and IM of PC specific NFAT5-KO compared to CTX and IM from control mice. PC specific loss of NFAT5 in the renal CTX increased the expression of 2260 transcripts, whereas 1224 transcripts were reduced compared to control CTX (Fig. 3D). Surprisingly, loss of NFAT5 function resulted in 4552 transcripts being increased in expression in the IM, whereas only 686 transcripts had reduced expression (Fig. 3E). A complete list of differentially expressed genes is provided as supplementary data (Supplemental Excel file 3).

**Figure 4:**
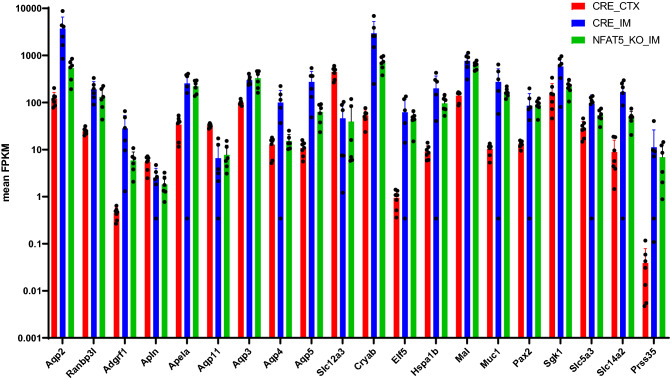
Expression level of selected osmo-regulated genes in cortex and inner medulla. Expression level (mean FPKM) of selected genes that are differentially expressed between the cortex (CTX) and inner medulla (IM) of control (CRE) kidneys. The green bars show the expression level of these transcripts in the IM of principal cell specific NFAT5 deficient mice (NFAT5_KO_IM).

We next analyzed the expression pattern of the osmoregulated transcripts (depicted in Fig. 2C). The mean expression values (FPKM) of these transcripts in the CTX and IM of control and NFAT5-KO mice is shown in Fig. 4. Similar to the results obtained with the primary cultivated IMCD cells, the expression of common hypertonicity regulated transcripts is higher in the IM compared to CTX and loss of NFAT5 expression in the PC cells is associated with reduced expression of the majority of these transcripts. These results recapitulates those obtained from primary cultivated IMCD cells and shows that NFAT5, either in vitro (cell culture conditions) or in vivo (IM vs. CTX) is a master regulator in expression of hypertonicity induced transcripts. For selected genes, changes in expression level were validated by real time PCR (Supplemental Fig. 5).

### Novel NFAT5 target genes

**Figure 5:**
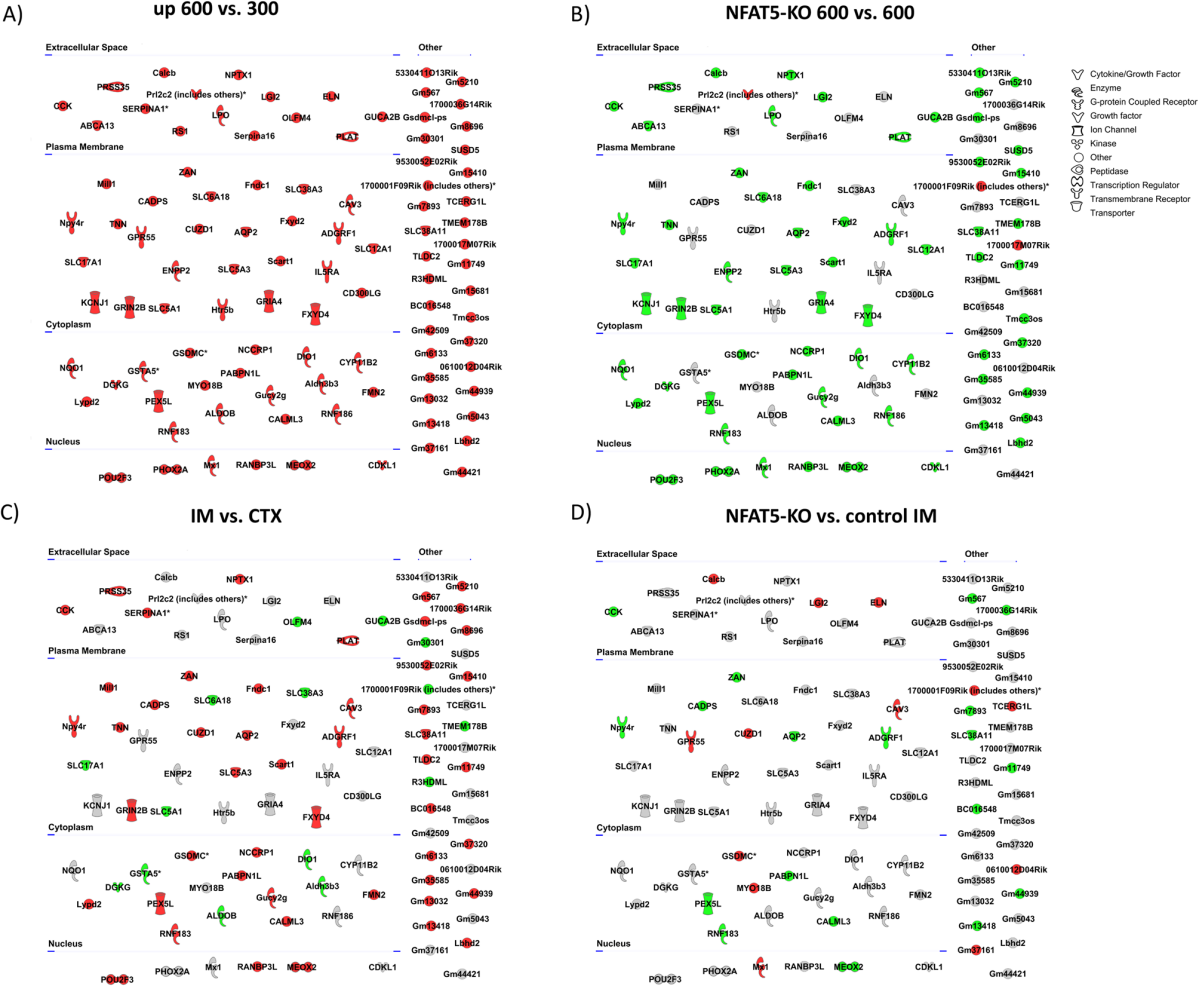
Top 100 genes increased under hypertonic conditions in IMCD cells. (**A**) Top 100 up regulated transcripts and their predicted subcellular localization in primary IMCD cells cultivated at 600 mosmol/kg compared to control conditions (300 mosmol/kg). (**B**) Changes in expression level in NFAT5-KO cells compared to control cells, cultivated at 600 mosmol/kg. (**C**) observed changes in renal IM vs. CTX. (**D**) Changes between NFAT5-KO IM vs control IM. The color shows the direction of regulation (green = decreased with log2 fold change < − 1; red = increased with log2 fold change > 1). Transcripts that did not met the criteria are labeled in grey.

**Figure 6:**
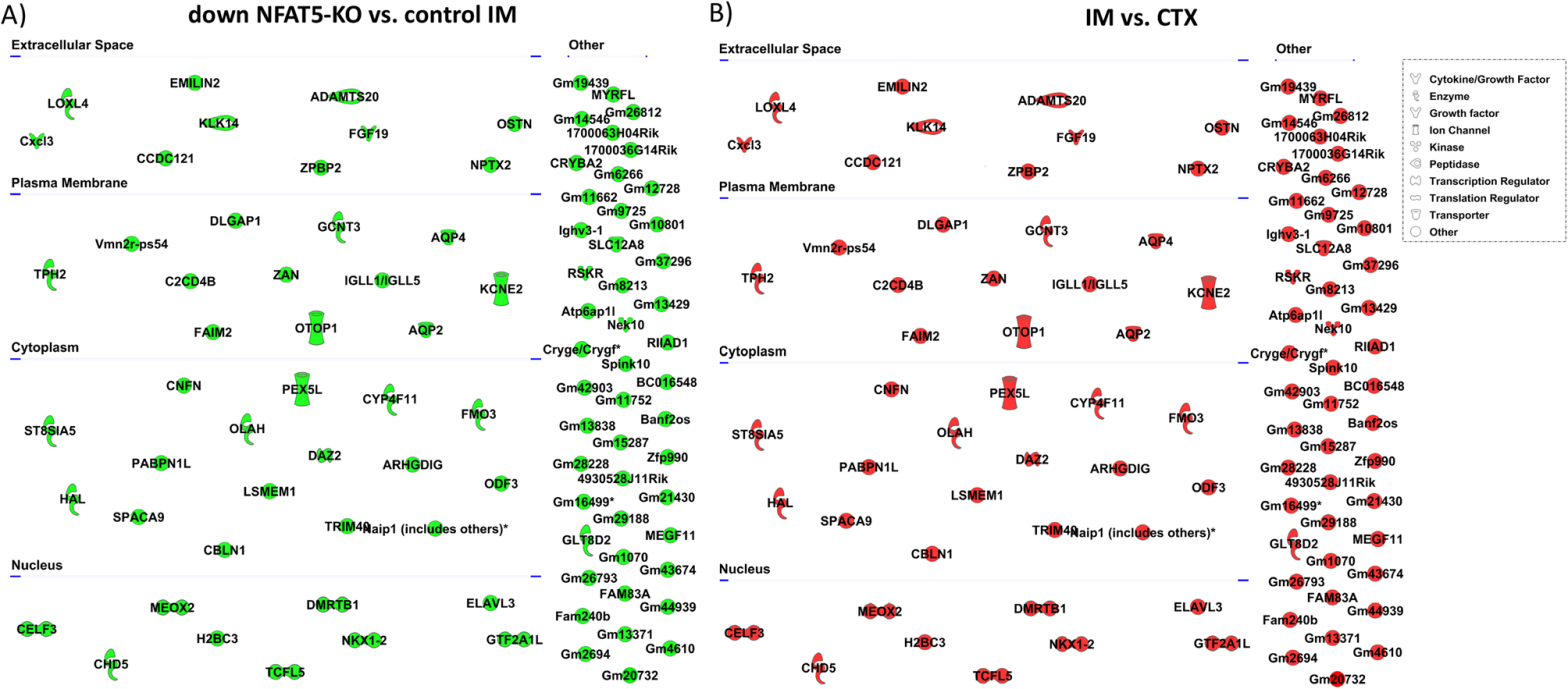
Top 100 decreased genes in NFAT5-KO IM. (**A**) The top 100 down regulated transcripts in NFAT5-KO IM compared to control IM. (**B**) Changes in expression level in control IM compared to control CTX. The color shows the direction of regulation (green = down regulated; red = up regulated). Color intensity indicates the relative differences in expression, with stronger staining marking a difference of a log2 fold change of − 1/1 or greater.

To identify and visualize additional NFAT5 target genes we used the top 100 transcripts increased by hypertonicity in primary cultivated IMCD cells (Fig. 5A) and mapped, using Ingenuity Pathway Analysis (IPA), their changes in expression in NFAT5-KO cells, in renal IM vs. renal CTX and in renal IM of NFAT5-KO vs control IM. Nearly all of these transcripts showed reduced expression in NFAT5-KO cells (Fig. 5B). In addition, approximately 2/3 of these transcripts were increased in expression in renal IM compared to renal CTX, including *Prss35, Meox2, Adgrf1, Fxyd4, Npy4r, Aqp2, Rnf183* and *Pex5l* (Fig. 5C)*.* The expression level of these transcripts were reduced in NFAT5-KO kidneys (Fig. 5D), indicating that their expression is regulated by the action of NFAT5. A similar analysis was performed using the top 100 decreased transcripts in NFAT5-KO IM vs. control. *Nek10* (log2 fold change 5.3), *Fgf19* (log2 fold change 4.8) and *Fam83a* (log2 fold change 3.8) were the transcripts with the greatest reduction in expression after NFAT deletion (Fig. 6A). When comparing the expression levels of this gene set in control CTX vs. control IM, all were increased in expression in IM relative to CTX (Fig. 6B) indicating that their expression is induced by hypertonicity and the activation of NFAT5.

### Identification of transcriptional networks

**Figure 7:**
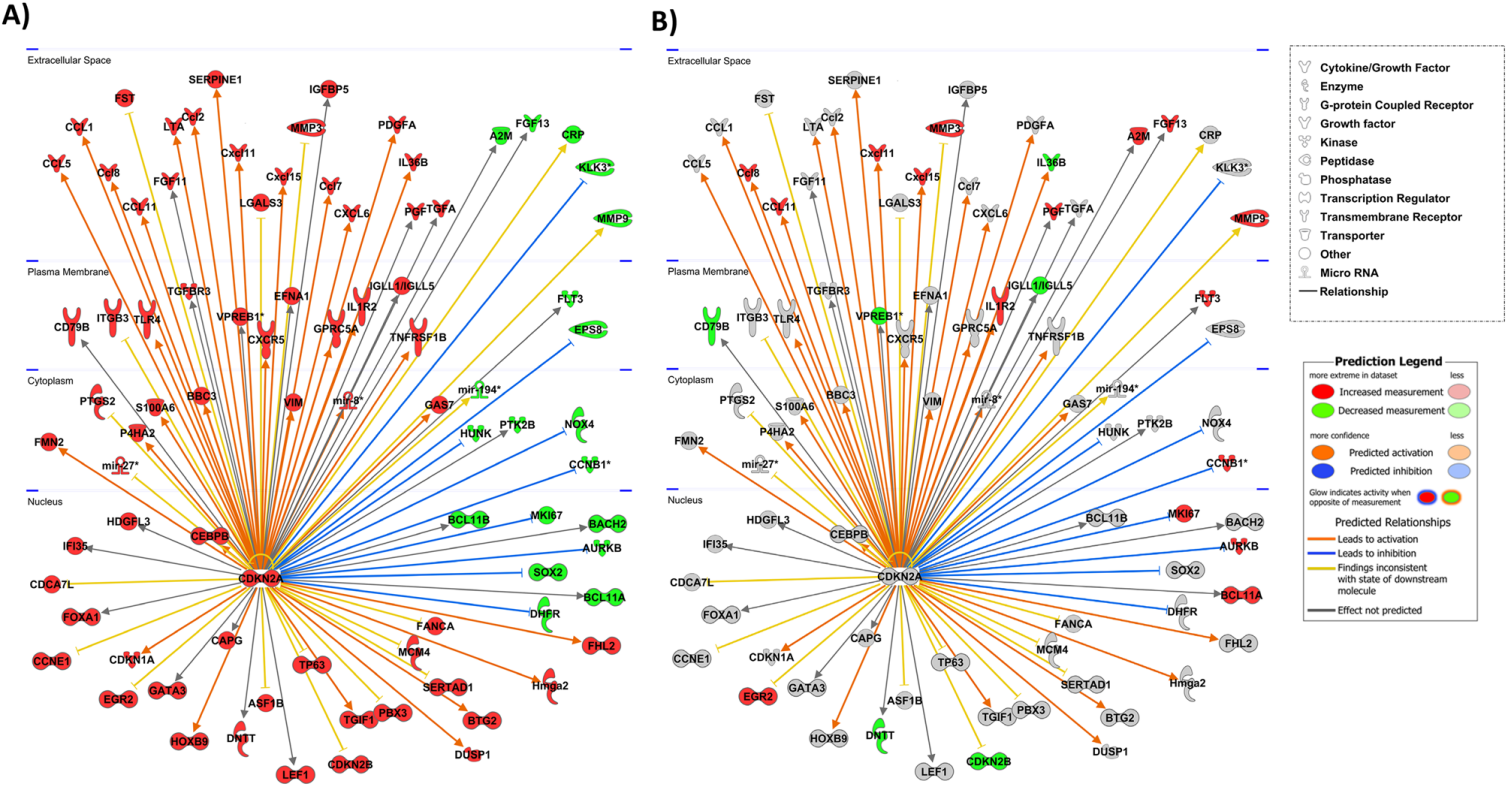
Activation of CDKN2A and associated network. Functional analysis using Ingenuity’s pathway analysis (IPA) predicted activation of CDKN2A. (**A**) Shows downstream transcripts that are deregulated in IM vs CTX and the direction of deregulation, predicting activation of CDKN2A in the IM. (**B**) Based on the expression pattern, the level of activation id disturbed in NFAT-KO IM vs. control IM. Red and green shaped molecules indicate increased and decreased expressions, respectively. The orange/red lines indicate activation, the blue lines indicate inhibition, and yellow lines indicate findings inconsistent with the state of downstream activity. The grey lines indicate that the effect was not predicted.

**Figure 8:**
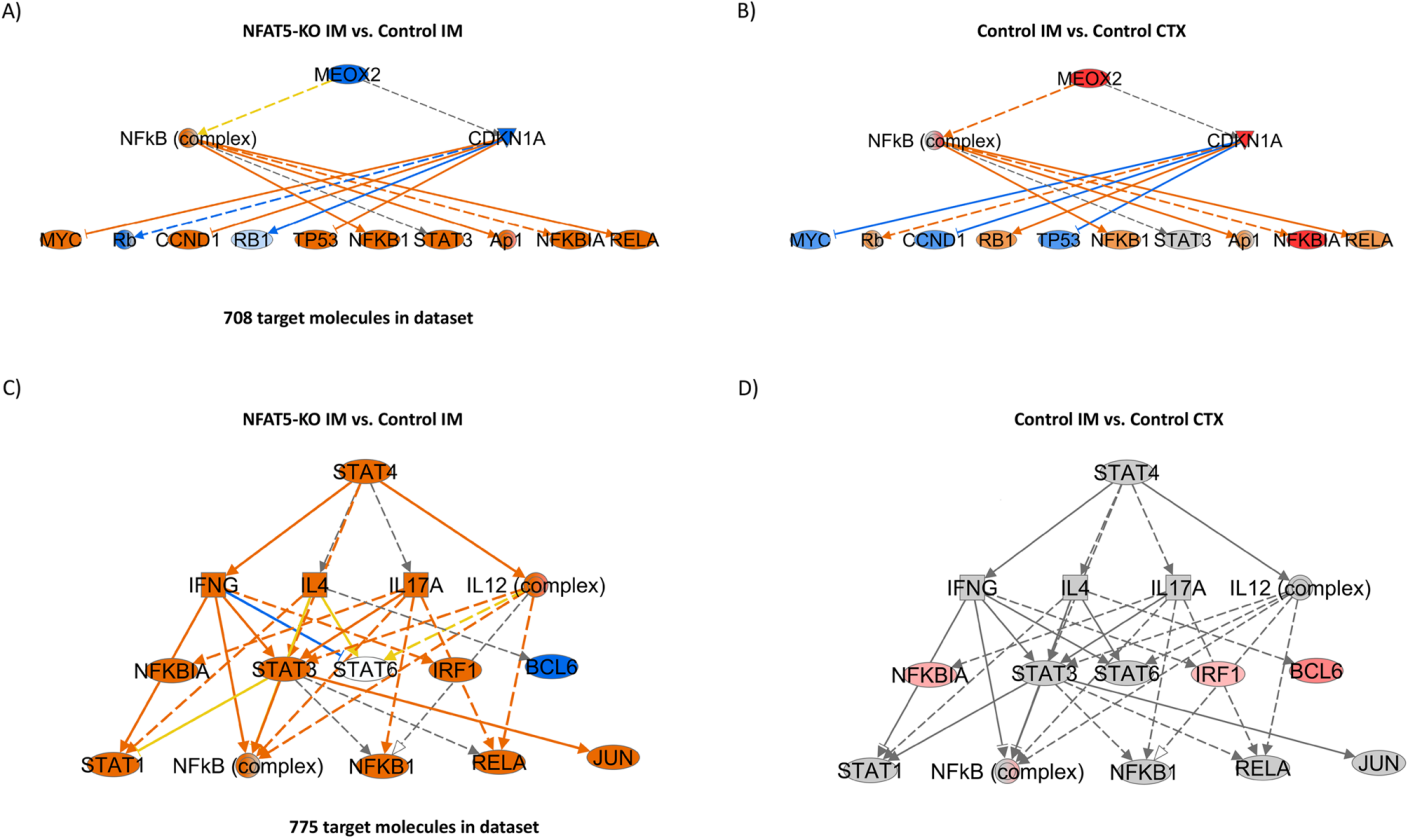
Loss of NFAT5 in IM is predicted to inhibit MEOX2 and activation of a STAT4 mechanistic network. Functional analysis using Ingenuity’s pathway analysis (IPA) predicted activation of MEOX2 and associated molecules. (**A**) MEOX2 and associated network predicted to be affected based on the gene expression pattern in NFAT5-KO IM compared to control IM. (**B**) Network and the predicted activation state based on the gene expression pattern obtained in control IM vs CTX comparison. (**C**,**D**) The predicted activation of STAT4 mechanistic network based on changes in gene expression in NFAT5-KO IM vs control and control IM vs CTX, respectively. The colors indicate predicted activation state. Orange lines/shapes = predicted activation/activated; blue lines/shapes = predicted inhibition/inhibited.

Hypertonicity affects the expression of genes coding for transcription factors like *Elf5, Pax2* or the mesodermal transcription factor *Meox2* that also could contribute to the observed differences in gene expression. NFAT5 also regulates the expression of *Pax2, Meox2* and other transcription factors*.* Thus, we used IPA to identify transcripts coding for transcriptional regulators that were altered between control renal IM and CTX that could contribute to the observed differences in gene expression pattern. 132 transcripts coding for transcriptional regulators were differentially expressed (104 increased and 28 decreased with a log2 FC > − 1/1). Since differences in expression level alone are not sufficient to predict activation of a certain factor, we performed transcription regulator activity analysis using IPA. Out of the 132 differentially expressed transcriptional regulators, 30 showed alterations in their predicted activation state (9 predicted to be inhibited, 21 predicted to be activated) between renal IM and CTX (Supplemental Excel file 4). Compared to renal CTX the top inhibited transcriptional regulators in the renal IM were *Hnf1a, Lhx1, Cbx4* and *Hnf4a*, whereas the top activated regulators include *Rela*, Hif1a and *Cdkn2a. Cdkn2a* expression in renal IM is higher (Log2 fold change 2.9) compared to CTX and Fig. 7A shows the predicted influence of *Cdkn2a* on 85 differentially expressed transcripts. The mapping of differentially expressed genes in NFAT5-KO IM vs. control IM identified 22 common transcripts, the majority of which had an inverse expression pattern relative to controls (Fig. 7B). The lists of the altered genes are provided as supplemental data (Supplemental Excel file 5). The same type of analysis was performed with the differentially expressed genes between NFAT5-KO IM and control IM. There were 33 transcriptional regulators predicted to be activated and 14 to be inhibited. One of the transcription factors predicted to be inhibited was *Meox2*. *Meox2* is decreased in NFAT5 deficient mice and primary IMCD cells (Fig. 2). Further analysis suggested several mechanistic networks are linked to *Meox2* inhibition. This network consists of 13 nodes including *Rela*, *Myc* or *Ccnd1* that regulate the expression or activity of 708 differentially expressed transcripts in NFAT5-KO IM compared to control IM. Figure 8A shows the connection of the 13 nodes and their predicated state of activation. The predicted activation of this network or members of this network is decreased or even inversed in control IM vs. CTX (Fig. 8B). Similar to MEOX2, loss of NFAT5 function in renal IM is associated with predicted activation of transcription factors and mechanistic networks compared to control IM. Figure 8C shows as an example STAT4 and the mechanistic network with STAT4 as the hub. This network consists of 15 nodes including IFNG, IL4, IRF1 or BCL6, and it has an influence on the activity of 775 transcripts that are differentially expressed in NFAT5-KO IM vs. control IM. BCL6 is the only node member that’s predicated to be inhibited in NFAT5 deficient IM (Fig. 8C). The activity of BCL6 is predicted to be activated in IM of control kidneys compared to control CTX, while the majority of the other members of this network showed no predicted activation or inhibition indicating that these changes are associated with loss of NFAT5 function (Fig. 8D). Together, these data indicate that several transcription regulators might be involved in the observed differences in gene expression between renal IM and CTX and that loss of NFAT5 function has an influence on these transcriptional regulators and networks.

### Functional analysis indicates inflammatory responses in NFAT5-KO mice

**Figure 9:**
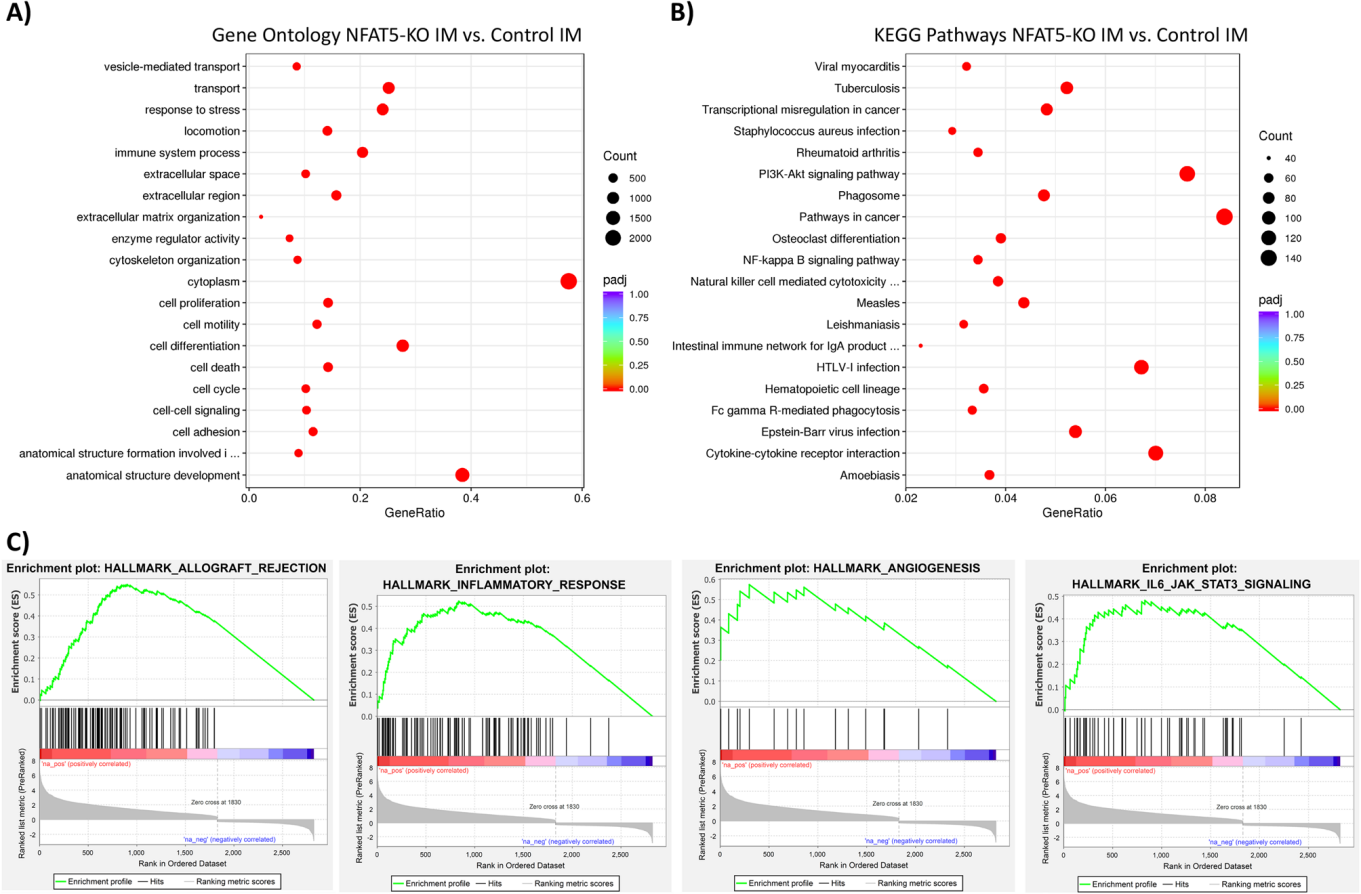
Enriched gene ontology terms, Enriched KEGG signaling pathways and gene set enrichment analysis of differentially expressed genes in NFAT5-KO IM vs. control IM. Differentially expressed genes in inner medulla of NFAT5-KO IM compared to control IM were used for functional annotation using gene ontology terms (GO). (**A**) The top 20 statistically enriched GO terms. (**B**) The top 20 KEGG signaling pathways associated with the differentially expressed genes. (**C**) The top 4 enriched gene sets from the differentially expressed genes using GSEA.

The predicted activation of *Stat4* and associated network members like *Ifng, Il4* or *Irf1* suggests increased expression of immune system associated genes. Indeed, further analysis of the expression data suggests that the kidneys of NFAT5-KO mice are affected by inflammation. For example, increased expression of *Cd4*, *Cd8* or *Cd14* indicates infiltration of immune cells in the kidneys of NFAT5-KO mice. Functional analysis of the differentially expressed genes by gene ontology (GO) terms and KEGG signaling pathways further supports this. In NFAT5 deficient mice, expression of genes classified to be involved in GO terms “immune system process”, “cell adhesion”, “response to stress” or “transport” are enriched in the renal IM compared to CTR mice (Fig. 9A). KEGG pathway analysis showed enrichment of differentially expressed genes associated for example in “Natural killer cell mediated cytotoxicity”, “Cytokine-cytokine receptor interaction” or “Fc gamma R-mediated phagocytosis” and other signaling pathways (Fig. 9B). Same analysis were performed for differentially expressed between control CTX and NFAT5-KO CTX (Supplemental Fig. 6).

The lists of differentially expressed genes in NFAT5-KO kidneys were used for gene set enrichment analysis (GSEA) using hallmark gene sets^32,33^. In the IM of NFAT5-KO kidneys, upregulated genes were enriched in the Hallmark gene sets “Allograft Rejection”, “Inflammatory Response” or “IL6_JAK_STAT3_Signaling” (Fig. 9C). Similar genes sets were enriched when using the list of differentially expressed genes between CTX of control and NFAT5-KO mice (Supplemental Fig. 7).

### Loss of NFAT5 induces a kidney injury associated gene expression profile

**Figure 10:**
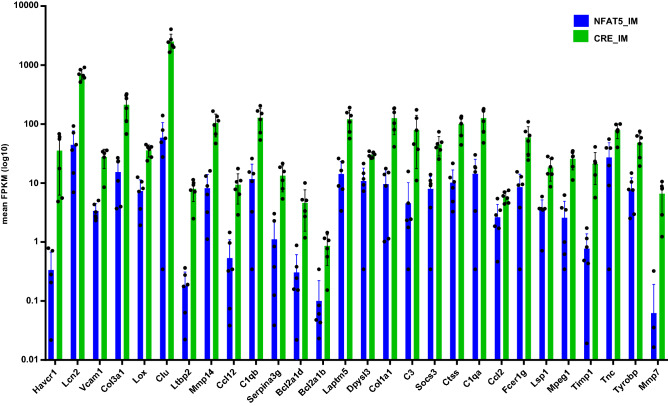
Expression of kidney injury marker genes are increased in NFAT5-KO IM. The expression level (mean FPKM) of well characterized kidney injury marker genes that are differentially expressed between control IM (blue) and NFAT5-KO IM (green).

Loss of NFAT5 induced expression of kidney injury markers. For example, the expression of lipocalin-2 (*Lcn2*, log2 fold change 5.0 in CTX and 4.0 in IM) and kidney injury molecule (*KIM1* or *Havcr1*, log2 fold change 6.8 in CTX and 6.6 in IM) were greatly increased after the loss of NFAT5 expression in PCs. The large increase in *Lcn2* and *Havcr1* expression combined with the reduced renal function^31^ suggests that loss of NFAT5 is associated with a kidney injury like phenotype. To examine this, we assessed if 51 kidney injury associated genes^34–36^ and Mmp7^37^ (also associated with kidney injury) were differentially expressed in a NFAT5 dependent manner. We used only genes that were differentially expressed both in CTX and IM of NFAT5 mice and showed at least a log_2_ fold change of 2 or higher. Using these criteria, 28 genes (including Mmp7) showed increased expression in NFAT5-KO mice compared to corresponding control mice (Fig. 10). For selected transcripts the changes in expression level were validated by real time PCR (Supplemental Fig. 8).Together these results indicate that loss of NFAT5 function recapitulates a kidney injury like phenotype.

## Discussion

The renal corticomedullary osmotic gradient is essential for the generation of a concentrated urine. Under cell culture conditions, hypertonicity induces the expression of kidney specific transcripts. In the kidneys, the osmotic gradient allows a spatial expression pattern of genes that are not present in most other cell types. The factors that contribute to this gene expression pattern are not known. To investigate these mechanisms in greater detail, here we performed large scale transcriptional profiling of osmolality induced gene expression changes in the kidney and examined the role of NFAT5. Our data demonstrate that; (1) NFAT5 is the master transcription factor for hypertonicity-induced gene expression, (2) NFAT5 plays an important role in gene expression independently of hypertonic stress, and (3) lack of NFAT5 in vivo results in responses associated to kidney injury. In the following we discuss our findings in respect to previous studies and the overall implications of our results for understanding the role of NFAT5 in gene expression.

Our previous microarray analysis of primary rat IMCD cells cultivated in different osmolality medium demonstrated the broad effect of hypertonicity on gene expression, with large increases in expression of e.g. *Aqp2, Slc14a1, Prss35 or Ranbp3l*^9^. Here, we used primary mouse IMCD cells and again after cultivation under hypertonic conditions there were large changes in gene expression of *Aqp2, Slc14a1, Prss35* and *Ranbp3l*. A direct role for NFAT5 in modulating expression of e.g. *Aqp2, Ranbp3l* or *Rnf183* has been shown in different studies using targeted approaches^23,25,38–40^. However, assessing the global role of NFAT5 has been hampered, as isolating primary cultured IMCD cells from NFAT5-deficient mice is constrained by the renal atrophy and survival rate of the mice^25^, and genetic manipulation of primary cultures is technically challenging. Therefore, to address this challenge we isolated primary IMCD cells from the kidneys of mice with tamoxifen inducible *Nfat5* deletion^28^ and treated them in vitro with tamoxifen, allowing us to examine direct effects of NFAT5 deletion on gene expression. In these cells, loss of NFAT5 function was associated with reduced expression of more than 200 transcripts. The majority of these transcripts were also reduced in the IM of NFAT5-KO mice, highlighting the broad role of NFAT5 as a key transcription factor in the kidney. Reduced expression was observed for *AQP2-4*, *Slc14a2*, *Slc5a3 or Hspa1b,* transcripts coding for proteins involved in urine concentration or adaptation to hypertonic stress.

Beside these known NFAT5 targets, several novel NFAT5 target genes were identified. For example the expression of *Gsdmc2-4* was reduced in NFAT5 deficient cells. These transcripts are coding for the gasdermin protein family members which in the kidney have the highest expression in the IM and are involved in programed cell death and inflammation^29^. Another example of a novel NFAT5 regulated gene is *Apela,* which encodes the Apelin receptor early endogenous ligand (APELA). APELA and Apelin (APLN) are ligands of the Apelin receptor^41^, both of which are involved in urine concentration^42^. External application of APELA antagonizes the effects of AVP^42^ and causes aquaresis in rodents^43^, at least in part due to inhibition of AQP2 expression and trafficking^44^. Our data suggests that NFAT5 induced increases in *Apela* may be a mechanism to counteract the prolonged actions of AVP and limit long-term exposure of IM cells to hypertonicity. APELA also protects against acute kidney injury^45^ improves cardio-renal outcome after septic shock^46^, lowers blood pressure by antagonizing the renin-angiotensin system^47^, has a tumor suppressor function in renal cancer^48^ and is a prognostic marker for patients with diabetic nephropathy^49^. In all of these situations high levels of APELA are associated with increased renal function. Our data indicate that the IM could be an endogenous source for APELA and that the NFAT5 driven increases in *Apela* may contribute to these beneficial effects.

Over 10,000 genes were differentially expressed between renal CTX and IM, with a larger number of transcripts (> 6000) showing increased in expression in the IM compared to CTX. By comparing the data, many of the transcripts with the largest hypertonicity induced changes in expression were localized to renal inner medullary segments, which are challenged by the greatest hypertonic environment. These differences in gene expression between cortex and medulla were also present in the NFAT5-KO mice, yet the ratio between up and down regulated genes increased from around 2:1 in control mice to approximately 3.5:1 in the NFAT5-KO mice. This is surprising as the NFAT5 mice cannot generate a hyperosmotic medullary interstitium and suffer from NDI^31^, so we expected more genes with reduced expression due to loss of function and a lack of osmotic difference between the two regions. However, what this data suggests is that NFAT5 is not only important for regulating gene expression in the face of hyperosmotic stress, but it also modulates gene transcription throughout the collecting duct.

The reduction in *Aqp2* expression observed in vivo was not as large as that in primary IMCD cells lacking NFAT5. Furthermore, although hyperosmolality induced changes in gene expression for the majority of transcripts were severely blunted after NFAT deletion in vitro or in vivo, they were not fully prevented. Together this emphasizes that other factors besides NFAT5 contribute to the (hypertonicity induced) gene expression. One of these may be the E74 Like ETS Transcription Factor 5 (*Elf5*), expression of which is increased by hypertonicity^9^. *Elf5* is specifically localized to the PC of the collecting duct and contributes to the expression of *Aqp2* and *Avpr2*^50^. As loss of NFAT5 has only a minor effect on *Elf5* expression, the actions of *Elf5* might contribute to the remaining *Aqp2* expression, but alone it cannot compensate for the loss of NFAT5. The transcription factor *Pax2 *(encoding PAX2)*,* which promotes tolerance to hypertonicity^51^, was also reduced after NFAT5 deletion. PAX2 is important for epigenetic regulation of vasopressin receptor (*Avpr2*) expression^52^, and PAX2 deficient mice having a urinary concentrating defect alongside reduced aquaporin and urea transporter expression^53^. These mice also have reduced expression of *Fxyd4*, *Prss35, Apela, Rnf183 or Adgfr1*, similar to what we observed after NFAT5 deletion. Hence, some of the actions of NFAT5 may be mediated via PAX2.

Loss of NFAT5 was predicted to activate different transcriptional networks like STAT4 and STAT4 associated mechanistic networks (including STAT1, STAT3, NFKB1 or IRF1) and may contribute to the altered expression of various genes. STAT3, which is activated under pathological conditions and contributes to kidney injury, is a promising target for treatment of kidney diseases^54^. Together with STAT4, the transcriptional factor interferon regulatory factor 1 (IRF1) is also predicted to be activated. IRF1 activity is associated with cardio-renal syndrome type 4^55^ and promotes renal fibrosis by reducing klotho^56^. As STAT3, IRF1 and NFKB1 are drivers of kidney fibrosis^57^, they could be involved in the kidney injury related transcriptional profile after loss of NFAT5.

Further analysis indicated that loss of NFAT5 induces a general renal injury/inflammation like phenotype, suggesting a novel previously unknown role of NFAT5. The protective role of NFAT5 is also described in other studies^58–60^. In line with this our data show an increased expression of transcripts like *Lcn2* (encoding Lipocalin 2 or neutrophil gelatinase-associated lipocalin, NGAL) and *Havcr1*, classical biomarkers for kidney injury. The expression of these factors were greatly increased in both the renal cortex and medulla of PC specific NFAT5-KO mice. Compared with that, hypertonicity reduces *Lcn2* expression and secretion in primary IMCD cells^61^ and mCCD(cl1) cells^62^, possibly explaining why loss of NFAT5 function is associated with increased *Lcn2* expression in vivo. Interestingly, expression of *Lcn2* is increased in PAX2/PAX8 deficient mice underlining our hypothesis that besides NFAT5 other factors contribute to the observed changes in gene expression and kidney function^53^. However, the findings described here that loss of NFAT5 function increases expression of kidney injury markers indicates that not only NFAT5 plays a protective role against kidney injury, but may also be a central hub in controlling expression of kidney injury molecules. Although loss of NFAT5 reduces the osmoprotective capacity of PCs and could lead to cell apoptosis and an inflammatory response, this cannot fully explain the findings as similar responses are observed in the cortex of PC specific NFAT5-KO mice that would not be expected to be affected by osmolality. Further analysis and mechanistic studies are required to identify how NFAT5 loss induces the kidney injury/inflammatory phenotype.

In summary, this is the first study using in vitro and in vivo models to show the extensive contribution of NFAT5 to hypertonicity and non-hypertonicity induced gene expression. NFAT5 expression drives a transcriptional network that is a major contributor to gene expression in the collecting duct PC. Hence, NFAT5 can be considered a master regulator of renal function. Furthermore, the inflammation and kidney injury-like phenotype observed following NFAT5 deletion suggests that NFAT5 activity contributes to various pathophysiological conditions.

## Methods

### Cell culture

All methods were performed in accordance with the relevant guidelines and regulations.

Primary mouse IMCD cells were prepared from mice kidneys as described before^39,63^. The cells were seeded in plates coated with collagen type IV (10376931, Thermo Fischer Scientific, Waltham, Massachusetts, United States) and cultivated in DMEM (FG 0435, Biochrom, Berlin, Germany) containing 1% penicillin and streptomycin, 1% non-essential amino acids (11140050, Thermo Fischer Scientific), and 1% Ultroser G (15950-017, CytoGen GmbH, Wetzlar, Germany). All cells were cultured at 37 °C and 5% CO_2_. The medium osmolality was adjusted to 600 mosmol/kg by the addition of 100 mM NaCl (71376, Sigma Aldrich) and 100 mM urea (U5378, Sigma Aldrich) to the corresponding medium.

### Immunofluorescence

Immunofluorescence was performed as described before^64^. Cells were seeded in 24-well plates on glass cover slips. Medium was then removed, and cells were fixed in 4% formalin. Unspecific binding sites were blocked by incubation with fish skin gelatin (0.3% in PBS, G7765-1L, Sigma Aldrich). Primary antibodies were applied in gelatin solution and incubated at 37 °C for 1 h. Three wash steps (15 min) were performed with PBS and the cells were incubated for 1 h with the secondary Alexa-labeled antibody solution in PBS. The cell nucleus was stained with 4′,6-diamidino-2-phenylindole (DAPI, 268298, Merck Millipore, Burlington, Massachusetts, USA). Cells were washed three times with PBS (15 min) and mounted on glass slides with Fluoroshield histology mounting medium (F6182-20 ml, Sigma Aldrich). Images were taken on a Keyence BZ-8100E microscope (Keyence Corporation, Osaka, Japan). We used the following antibodies: anti-NFAT5 rabbit (ab3446, Abcam, Cambridge, UK) and Goat anti-Rabbit IgG (H + L) Alexa Fluor 488 (A-11034, Thermo Fisher Scentific).

### Conditional ex-vivo NFAT5 knockout in IMCD cells

The use of mice in this study was performed in in accordance with ARRIVE guidelines. Breeding of transgenic mice and use of mice for generation of primary IMCD cells was approved by institutional committee (approval ID 2-1482 MLU) of the Martin-Luther-University Halle-Wittenberg, Halle Germany. *Nfat5*^fl/fl^-Ubc-*Cre*-ERT2^+/−^ mice were kindly provided by the group of Prof. Christoph Küper*.* In these mice exon 4 of the *Nfat5* allele is flanked by *LoxP* sites^28^. Further, these mice harbor a derivative of the *Cre*-recombinase, which is under the control of the ubiqitin-C promoter (Ubc-Cre-ERT2). The genotyping of the mice was performed as described before^28^. From these *Nfat5*^fl/fl^-Ubc-*Cre*-ERT2^+/−^ mice we prepared primary cultured IMCD cells. The cells were seeded in 24 well plates and after 48 h the cells were treated for 24 h with 1 µg/ml 4-hydroxytamoxifen (4-OH-TM) (T176-10MG, Sigma Aldrich), followed by an additional 3 days of cultivation either at 300 or 600 mosmol/kg.

### Principal cell specific NFAT5 knock out

To obtain mice that are deficient for NFAT5 in the PC of the collecting duct we crossed floxed NFAT5 mice (NFAT^fl/fl^) with Aqp2-Cre mice, which have CRE recombinase expression under the control of the Aqp2 gene promotor^65^. The Aqp2-Cre deleter mice were kindly provided by Dr. Juliette Hadchouel. Mice genotyping was performed as described^28,66^. Since the insertion of the Cre recombinase destroys one allele of the *Aqp2* gene, we used only NFAT5^fl/fl^-AQP2-CRE^+/−^ mice, with Aqp2-Cre^+/−^ mice as controls. We used these mice to prepare primary IMCD cells. To analyze the contribution of NFAT5 on gene expression in vivo, we isolated total RNA from renal cortex (CTX) and renal medulla (including papilla, IM) using the Ribopure kit (Invitrogen, Carlsbad, CA, USA) and performed gene expression profiling by Next Generation Sequencing (NGS).

### Preparation of samples for NGS

For gene expression analysis using NGS RNA-sequencing (RNA-seq), total RNA from primary IMCD cells was isolated using the Gen Elute Mammalian Total RNA prep kit (RTN350-1KT, Sigma Aldrich) and subjected to next generation RNA sequencing. Total RNA from renal cortex and renal inner medulla was performed with the RiboPure kit (Ambion) according to the manufacturer’s instructions. The quality control, sequencing and bioinformatics were performed as described^67^. The data presented here and the raw data are available via Gene Expression Omnibus (Acc. No. GSE195881).

### RNA quantification and qualification

RNA degradation and contamination was monitored on 1% agarose gels. RNA purity was checked using the NanoPhotometer spectrophotometer (IMPLEN, CA, USA). RNA integrity and quantitation were assessed using the RNA Nano 6000 Assay Kit of the Bioanalyzer 2100 system (Agilent Technologies, CA, USA). Only samples with a RNA integrity number (RIN) of > 7 were used for analysis. One sample did not meet the criteria.

### Library preparation for transcriptome sequencing

A total amount of 1 μg RNA per sample was used as input material for the RNA sample preparations. Sequencing libraries were generated using NEBNext UltraTM RNA Library Prep Kit for Illumina (New England Biolabs, Ipswich, MA, USA) following manufacturer’s recommendations and index codes were added to attribute sequences to each sample. Briefly, mRNA was purified from total RNA using poly-T oligo-attached magnetic beads. Fragmentation was carried out using divalent cations under elevated temperature in NEBNext First Strand Synthesis Reaction Buffer (5X). First strand cDNA was synthesized using random hexamer primer and M-MuLV Reverse Transcriptase (RNase H-). Second strand cDNA synthesis was subsequently performed using DNA Polymerase I and RNase H. Remaining overhangs were converted into blunt ends via exonuclease/polymerase activities. After adenylation of 3′ ends of DNA fragments, NEBNext Adaptor with hairpin loop structure were ligated to prepare for hybridization. In order to select cDNA fragments of preferentially 150–200 bp in length, the library fragments were purified with AMPure XP system (Beckman Coulter, Beverly, USA). Then 3 μl USER Enzyme (New England Biolabs, USA) was used with size-selected, adaptor ligated cDNA at 37 °C for 15 min followed by 5 min at 95 °C before PCR. Then PCR was performed with Phusion High-Fidelity DNA polymerase, Universal PCR primers and Index (X) Primer. At last, PCR products were purified (AMPure XP system) and library quality was assessed on the Agilent Bioanalyzer 2100 system.

### Clustering and sequencing

The clustering of the index-coded samples was performed on a cBot Cluster Generation System using PE Cluster Kit cBot-HS (Illumina, San Diego, CA, USA) according to the manufacturer’s instructions. After cluster generation, the library preparations were sequenced on an Illumina NovaSeq 6000 Sequencing System (read length: paired-end 150 bp).

### Data analysis and quality control

Raw data (raw reads) of FASTQ format were firstly processed through in-house scripts. In this step, clean data (clean reads) were obtained by removing reads containing adapter and poly-N sequences and reads with low quality from raw data. At the same time, Q20, Q30 and GC content of the clean data were calculated. All the downstream analyses were based on the clean data with high quality^68–70^.

### Mapping to reference genome

Reference genome and gene model annotation files were downloaded from genome website browser (NCBI/UCSC/Ensembl) directly. Paired-end clean reads were mapped to the reference genome using HISAT2 software^71^. HISAT2 uses a large set of small GFM indexes that collectively cover the whole genome. These small indexes (called local indexes), combined with several alignment strategies, enable rapid and accurate alignment of sequencing reads^72–74^. The data was then mapped on the GRCm38 (Mus musculus, Synoyms: mm10) genome.

### Quantification

HTSeq was used to count the read numbers mapped of each gene, including known and novel genes. And then RPKM of each gene was calculated based on the length of the gene and reads count mapped to this gene. RPKM, Reads Per Kilobase of exon model per Million mapped reads, considers the effect of sequencing depth and gene length for the reads count at the same time, and is currently the most commonly used method for estimating gene expression levels^75,76^.

### Differential expression analysis

Differential expression analysis between two conditions/groups (three biological replicates per condition) was performed using DESeq2 R package. DESeq2 provides statistical routines for determining differential expression in digital gene expression data using a model based on the negative binomial distribution. The resulting p values were adjusted using the Benjamini and Hochberg’s approach for controlling the False Discovery Rate (FDR). Genes with an adjusted *p* value < 0.05 found byDESeq2 were assigned as differentially expressed^77^.

### Enrichment analysis

A common way for searching shared functions among genes is to incorporate the biological knowledge provided by biological ontologies. Gene Ontology (GO) annotates genes to biological processes, molecular functions, and cellular components in a directed acyclic graph structure, and Kyoto Encyclopedia of Genes and Genomes (KEGG) annotates genes to pathway. KEGG is a database resource for understanding high-level functions and utilities of the biological system, such as the cell, the organism and the ecosystem, from molecular-level information, especially large-scale molecular datasets generated by genome sequencing and other high-through put experimental technologies (http://www.genome.jp/kegg/). We used KOBAS 2.0 to test the statistical enrichment of differential expression genes in KEGG pathways^78,79^.

### Identification of transcriptional networks

Ingenuity pathway analysis (IPA) was used to identify transcription factors (TFs) that were activated or inhibited based on changes in expression level of target genes in a given gene set. Based on the global differences in gene expression an activation z-score was generated, with a negative z-score predicting inhibition and a positive z-score suggesting activation of a transcriptional regulator. IPA was also used to identify a mechanistic network that could contribute to the observed differences.

## Supplementary Information

Supplementary Information 1.

Supplementary Information 2.

Supplementary Information 3.

Supplementary Information 4.

Supplementary Information 5.

Supplementary Information 6.

## Data Availability

The data presented here have been submitted to the public repository Gene Expression Omnibus (Acc. No. GSE195881; https://www.ncbi.nlm.nih.gov/geo/query/acc.cgi?acc=GSE195881).
