# Supplementary material for: The nuclear factor of activated T cells 5 (NFAT5) contributes to the renal corticomedullary differences in gene expression
Source: Sci Rep. 2022 Nov 24;12:20304. doi: 10.1038/s41598-022-24237-y (PMC9700710; doi:10.1038/s41598-022-24237-y)
Supplement: Supplementary file 6 — Supplementary Information 6. [file 41598_2022_24237_MOESM6_ESM.docx]

The nuclear factor of activated T cells 5 (NFAT5) contributes to the renal corticomedullary differences in gene expression

Dmitry Chernyakov^1^, Annika Fischer^1^, Max Brandau^1^, Federica Petrillo^2^, Robert A Fenton^2#^ and Bayram Edemir^1,3^*#

1. Department of Medicine, Hematology and Oncology, Martin Luther University Halle-Wittenberg, Halle (Saale), Germany
2. Department of Biomedicine, Aarhus University, Aarhus, Denmark
3. Institute for Physiology, Pathophysiology and Toxicology, Witten/Herdecke University, Witten, Germany

**Supplemetary Material**

Supplemental Figure 1: Spatial expression of potential NFAT5 regulated genes

Supplemental Figure 2: Spatial expression of common osmoregulated genes

Supplemental Figure 3: Spatial expression of genes that are deregulated in principal cell specific NFAT5-KO IMCD cells.

Supplemental Figure 4: Sex specific differences in gene expression in the renal cortex and renal inner medulla

Supplemental Figure 5: Validation for selected genes by real time PCR.

Supplemental Figure 6: Enriched gene ontology terms and Enriched KEGG signaling pathways associated with differentially expressed genes.

Supplemental Figure 7: Gene set enrichment analysis of differentially expressed genes in

NFAT5-KO cortex vs. control cortex

Supplemental Figure 8: Validation for selected genes by real time PCR.

Supplemental Table 1: RNA sequencing raw data

Supplemental Excel file 1: List of genes affected by hyperosmolality and loss of NFAT5 in primary cultivated IMCD cells

Supplemental Excel file 2: Venn diagram and list of common and unique genes from figure 2 b

Supplemental Excel file 3: List of differentially expressed genes between renal cortex and medulla and control and NFAT5-KO mice

Supplemental Excel file 4: List of affected transcription factors between renal cortex and medulla and between control and NFAT5-KO mice

Supplemental Excel file 5: List of CDKN2A target genes affected between renal cortex and medulla and between control and NFAT5-KO mice

**References**

**Supplemental Figure 1: Spatial expression of potential NFAT5 regulated genes.** We used the Kidney Cell Explorer ([1](#_ENREF_1)) to visualize the spatial expression pattern of common NFAT5 regulated genes in renal segments identified in Figure 2B. The numbers are coding for different cell types/segments as described in the legend.

**Supplemental Figure 2: Spatial expression of common osmoregulated genes.** We used the Kidney Cell Explorer to visualize the spatial expression pattern of osmolregulated genes. The numbers are coding for different cell types/segments as described in the legend.

**Supplemental Figure 3: Spatial expression of genes that are deregulated in principal cell specific NFAT5-KO IMCD cells.** A) Expression level (FPKM) of the top ten hypertonicity induced up-regulated transcripts and their expression level in PC specific NFAT5-KO IMCD cells. B) Venn diagram shows Identification of potential common NFAT5 target genes. C) Visualization of the spatial expression pattern of these genes using the kidney cell explorer. D) Effect of PC specific NFAT5-KO in IMCD cells on expression level of common osmoregulated transcripts.

**Supplemental Figure 4: Sex specific differences in gene expression in the renal cortex and renal inner medulla.** Volcano plots shows the number of differentially expressed genes in male renal cortex (mCRE_C) vs female cortex (fCRE_C, upper left panel) and male inner medulla (mCRE_M) vs female inner medulla (fCRE_M, upper right panel). The same analysis was performed for NFAT5 deficient mice (lower panel).

**Supplemental Figure 5: Validation for selected genes by real time PCR.** Changes in gene expression were validated for selected genes by real-time PCR in A) between renal CTX and renal IM in control kidneys and B) between renal IM of control and NFAT5-KO kidneys. Unpaired t test was performed for statistical analysis and significantly different expressed genes are marked by * (p < 0.05).Samples from three female and one male mice were used for analysis.

**Supplemental Figure 6: Enriched gene ontology terms and Enriched KEGG signaling pathways associated with differentially expressed genes.** Differentially expressed genes in cortex of NFAT5-KO were used for functional annotation using gene ontology terms (GO). The top 20 statistically enriched GO terms are shown in A). In the same way, the differentially expressed genes were used to identify KEGG signaling pathways with enriched genes. Panel B) shows the top 20 KEGG signaling pathways associated with differentially expressed genes.

**Supplemental Figure 7: Gene set enrichment analysis of differentially expressed genes in NFAT5-KO cortex vs. control cortex.** The ranked differentially expressed genes were used for gene set enrichment analysis. This figure shows the top six enriched gene sets. The complete list is provided as supplemental data.

**Supplemental Figure 8: Validation for selected genes by real time PCR.** Changes in gene expression were validated for selected genes that are up regulated in NFAT5-KO kidneys by real-time PCR in A) in the renal CTX between control and NFAT5-KO kidneys and B) in the renal MD between control and NFAT5-KO kidneys. Unpaired t test was performed for statistical analysis and significantly different expressed genes are marked by * (p < 0.05). Samples from three female and one male mice were used for analysis. MMP7 was only detectable in 1 CRE_IM samples and this samples served as reference for calculation.

**Table S1: RNA sequencing raw data**

| **sample** | **raw_**  **reads** | **clean**  **reads** | **raw_**  **data**  **(G)** | **total filtered**  **reads** | **total**  **Mapped reads** | **uniquely**  **mapped**  **reads** | **multiple mapped reads** |
| --- | --- | --- | --- | --- | --- | --- | --- |
| WT_300_1 | 30876766 | 30572023 | 9.3 | 61144046 | 58900644 (96.33%) | 56109926 (91.77%) | 2790718 (4.56%) |
| WT_300_2 | 25233293 | 24978521 | 7.6 | 49957042 | 48202603 (96.49%) | 46098484 (92.28%) | 2104119 (4.21%) |
| WT_600_1 | 31260122 | 30906540 | 9.4 | 61813080 | 59530190 (96.31%) | 56554604 (91.49%) | 2975586 (4.81%) |
| WT_600_2 | 23323269 | 23102680 | 7 | 46205360 | 44591991 (96.51%) | 42369059 (91.70%) | 2222932 (4.81%) |
|  |  |  |  |  |  |  |  |
| KO_300_1 | 27634448 | 27340212 | 8.3 | 54680424 | 52705328 (96.39%) | 50178212 (91.77%) | 2527116 (4.62%) |
| KO_300_2 | 26429916 | 26106464 | 7.9 | 52212928 | 50379037 (96.49%) | 47983263 (91.90%) | 2395774 (4.59%) |
| KO_600_1 | 34068738 | 33725632 | 10.2 | 67451264 | 64860709 (96.16%) | 61534271 (91.23%) | 3326438 (4.93%) |
| KO_600_2 | 21556787 | 21308711 | 6.5 | 42617422 | 41102552 (96.45%) | 38969735 (91.44%) | 2132817 (5.00%) |
|  |  |  |  |  |  |  |  |
| mICRE_C1 | 36087982 | 35660533 | 10.8 | 71321066 | 69675248 (97.69%) | 63651334 (89.25%) | 6023914 (8.45%) |
| fICRE_C2 | 42154697 | 41604314 | 12.6 | 83208628 | 81252398 (97.65%) | 74601276 (89.66%) | 6651122 (7.99%) |
| fICRE_C3 | 36432959 | 36042866 | 10.9 | 72085732 | 70465030 (97.75%) | 64617622 (89.64%) | 5847408 (8.11%) |
| fICRE_C4 | 46263425 | 45631584 | 13.9 | 91263168 | 89239453 (97.78%) | 81939628 (89.78%) | 7299825 (8.00%) |
| fICRE_C5 | 45496139 | 44665829 | 13.6 | 89331658 | 87211826 (97.63%) | 80068865 (89.63%) | 7142961 (8.00%) |
| mICRE_C6 | 45097809 | 44286508 | 13.5 | 88573016 | 86601825 (97.77%) | 79276462 (89.50%) | 7325363 (8.27%) |
| fICRE_C7 | 39227132 | 38665266 | 11.8 | 77330532 | 75506258 (97.64%) | 69498656 (89.87%) | 6007602 (7.77%) |
| mICRE_M1 | 40355916 | 39558483 | 12.1 | 79116966 | 77032758 (97.37%) | 70809737 (89.50%) | 6223021 (7.87%) |
| fICRE_M2 | 38540903 | 37900746 | 11.6 | 75801492 | 73808758 (97.37%) | 67660853 (89.26%) | 6147905 (8.11%) |
| fICRE_M3 | 34871748 | 34291511 | 10.5 | 68583022 | 66770239 (97.36%) | 63025105 (91.90%) | 3745134 (5.46%) |
| fICRE_M4 | 33451167 | 32892983 | 10 | 65785966 | 64107804 (97.45%) | 59123591 (89.87%) | 4984213 (7.58%) |
| fICRE_M5 | 36691894 | 36058482 | 11 | 72116964 | 69692077 (96.64%) | 64685601 (89.70%) | 5006476 (6.94%) |
| mICRE_M6 | 34535073 | 34018840 | 10.4 | 68037680 | 66339306 (97.50%) | 62084985 (91.25%) | 4254321 (6.25%) |
| fICRE_M7 |  |  |  |  |  |  |  |
| mNFA_C1 | 39921486 | 39292105 | 12 | 78584210 | 76578548 (97.45%) | 70645177 (89.90%) | 5933371 (7.55%) |
| mNFA_C2 | 32120366 | 31518162 | 9.6 | 63036324 | 61542423 (97.63%) | 56698198 (89.95%) | 4844225 (7.68%) |
| mNFA_C3 | 37381303 | 36714994 | 11.2 | 73429988 | 71637650 (97.56%) | 65720349 (89.50%) | 5917301 (8.06%) |
| fNFA_C4 | 38881610 | 38204286 | 11.7 | 76408572 | 74642770 (97.69%) | 69075255 (90.40%) | 5567515 (7.29%) |
| fNFA_C5 | 32657208 | 32129234 | 9.8 | 64258468 | 62760255 (97.67%) | 58411801 (90.90%) | 4348454 (6.77%) |
| fNFA_M6 | 31076514 | 30609432 | 9.3 | 84588318 | 82608335 (97.66%) | 76526733 (90.47%) | 6081602 (7.19%) |
| mNFA_M1 | 34603921 | 34099953 | 10.4 | 68199906 | 66465745 (97.46%) | 62830049 (92.13%) | 3635696 (5.33%) |
| mNFA_M2 | 40124489 | 39491026 | 12 | 73429988 | 71637650 (97.56%) | 65720349 (89.50%) | 5917301 (8.06%) |
| mNFA_M3 | 42860504 | 42143073 | 12.9 | 84286146 | 82096572 (97.40%) | 77809745 (92.32%) | 4286827 (5.09%) |
| fNFA_M4 | 40201535 | 39461649 | 12.1 | 78923298 | 76771035 (97.27%) | 72596373 (91.98%) | 4174662 (5.29%) |
| fNFA_M5 | 40898416 | 40299744 | 12.3 | 80599488 | 78467295 (97.35%) | 73063707 (90.65%) | 5403588 (6.70%) |
| fNFA_M6 | 31076514 | 30609432 | 9.3 | 61218864 | 59721775 (97.55%) | 56233035 (91.86%) | 3488740 (5.70%) |

sample: the names of samples

raw _reads: the original sequencing reads counts

clean_reads: number of reads after filtering

raw_data (G): raw reads number multiply read length, saved in G unit

total filtered reads: total number of filtered reads (Clean data).

total mapped: total number of reads that can be mapped to the reference genome.

uniquely mapped reads: number of reads that can be uniquely mapped to the reference genome.

multiple mapped reads: number of reads that can be mapped to multiple sites in the reference genome.

For quality control only samples with a RIN of >7 were used for analysis. One sample did not meet the criteria (fICRE_M7). The further RNA Sequencing was done by Novogene on an Illumina NovaSeq 6000 Sequencing System (read length: Paired-end 150 bp). The data was then mapped on the GRCm38 (*Mus musculus*, Synoyms: mm10) genome.

Reference List

1. Ransick, A., Lindström, N. O., Liu, J., Zhu, Q., Guo, J. J., Alvarado, G. F., Kim, A. D., Black, H. G., Kim, J., and McMahon, A. P. (2019) Single-Cell Profiling Reveals Sex, Lineage, and Regional Diversity in the Mouse Kidney. *Dev Cell* **51**, 399-413.e397
